# Influence of occlusal thickness on the fracture resistance of chairside milled lithium disilicate posterior full‐coverage single‐unit prostheses containing virgilite: A comparative in vitro study

**DOI:** 10.1111/jopr.13870

**Published:** 2024-05-24

**Authors:** Carlos A. Jurado, Christian Edgar Davila, Alexandra Davila, Alfredo I. Hernandez, Yukari Odagiri, Kelvin I. Afrashtehfar, Damian Lee

**Affiliations:** ^1^ Operative Dentistry Division Department of General Dentistry College of Dentistry University of Tennessee Health Science Center Memphis Tennessee USA; ^2^ Division of Prosthodontics Department of Restorative Dental Sciences University of Florida College of Dentistry Gainesville Florida USA; ^3^ Division of Prosthodontics, Department of Restorative Sciences School of Dentistry University of Alabama Birmingham Alabama USA; ^4^ A.T. Still University Arizona School of Dentistry and Oral Health Mesa Arizona USA; ^5^ Department of Operative Dentistry School of Dentistry Aichi Gakuin University Nagoya Japan; ^6^ Department of Reconstructive Dentistry and Gerodontology School of Dental Medicine Faculty of Medicine University of Bern Bern Switzerland; ^7^ Clinical Sciences Department College of Dentistry Ajman University Ajman City UAE; ^8^ Consultant Private Practice Limited to Prosthodontics and Pre‐Prosthetic Surgery Abu Dhabi UAE; ^9^ Consultant Private Practice Limited to Prosthodontics, Esthetic and Implant Dentistry Dubai UAE; ^10^ Division of Periodontology Rheinisch‐Westfälische Technische Hochschule (RWTH) Aachen University Hospital Aachen Germany; ^11^ Department of Prosthodontics Tufts University School of Dental Medicine Boston Massachusetts USA

**Keywords:** CAD‐CAM systems, computer‐aided design, dental crowns, dental materials, dental occlusion

## Abstract

**Purpose:**

To evaluate the fracture resistance of chairside computer‐aided design and computer‐aided manufacturing (CAD‐CAM) lithium disilicate mandibular posterior crowns with virgilite of different occlusal thicknesses and compare them to traditional lithium disilicate crowns.

**Materials and Methods:**

Seventy‐five chairside CAD‐CAM crowns were fabricated for mandibular right first molars, 60 from novel lithium disilicate with virgilite (CEREC Tessera, Dentsply Sirona), and 15 from traditional lithium disilicate (e.max CAD, Ivoclar Vivadent). These crowns were distributed across five groups based on occlusal thickness and material: Group 1 featured CEREC Tessera crowns with 0.8 mm thickness, Group 2 had 1.0 mm thickness, Group 3 had 1.2 mm thickness, Group 4 with 1.5 mm thickness, and Group 5 included e.max CAD crowns with 1.0 mm thickness. These crowns were luted onto 3D‐printed resin dies using Multilink Automix resin cement (Ivoclar Vivadent). Subsequently, they underwent cyclic loading (2,000,000 cycles at 1 Hz with a 275 N force) and loading until fracture. Scanning electron microscopy (SEM) assessed the fractured specimens. Statistical analysis involved one‐way ANOVA and the Kruskal‐Wallis Test (*α* = 0.05).

**Results:**

Fracture resistance varied significantly (<0.001) across mandibular molar crowns fabricated from chairside CAD‐CAM lithium disilicate containing virgilite, particularly between crowns with 0.8 mm and those with 1.2 and 1.5 mm occlusal thickness. However, no significant differences were found when comparing crowns with 1, 1.2, and 1.5 mm thicknesses. CEREC Tessera crowns with 1.5 mm thickness exhibited the highest resistance (2119 N/mm^2^), followed by those with 1.2 mm (1982 N/mm^2^), 1.0 mm (1763 N/mm^2^), and 0.8 mm (1144 N/mm^2^) thickness, whereas e.max CAD crowns with 1.0 mm occlusal thickness displayed the lowest resistance (814 N/mm^2^).

**Conclusions:**

The relationship between thickness and fracture resistance in the virgilite lithium disilicate full‐coverage crowns was directly proportional, indicating that increased thickness corresponded to higher fracture resistance. No significant differences were noted among crowns with thicknesses ranging from 1 to 1.5 mm. This novel ceramic exhibited superior fracture resistance compared to traditional lithium disilicate.

Lithium disilicate ceramics, initially introduced in 1988 as IPS Empress 2, have undergone significant advancements in composition and manufacturing methods.^1^, ^2^ These ceramics, classified as glass ceramics, found their early application in heat‐pressed restorations with a crystalline lithium disilicate filler content of around 70%, providing a flexural strength of 350 MPa.^3^, ^4^ In 2005, the introduction of IPS e.max Press marked an improvement in optical and mechanical features, achieving flexural strength values ranging from 370 to 460 MPa.^5^ This enhancement was attributed to the presence of elongated and closely interlocked disilicate crystals, inhibiting crack propagation.^6^ The integration of computer‐aided design and manufacturing (CAD‐CAM) technology into dentistry led to the development of ceramics compatible with these modern techniques. In 2006, IPS e.max CAD emerged as a CAD‐CAM‐specific lithium disilicate material, offering practitioners greater versatility.^7^

The lithium disilicate crystalline content undergoes different stages during the crystallization process, categorized into three phases.^8^, ^9^, ^10^, ^11^ The transformation concludes with lithium metasilicate crystals (blue stage) evolving into lithium disilicate crystals. The current CAD‐CAM lithium disilicate version (e.max CAD by Ivoclar Vivadent), featuring 0.2−1.0‐micron platelet‐shaped crystals, is provided in partially crystallized blocks. These blocks come in a range of shades and translucency levels and require full crystallization in a furnace for clinical application to achieve the desired shade and translucency.^8^ With approximately 40% lithium metasilicate crystals (Li_2_SiO_3_) embedded in a glassy phase alongside lithium disilicate nuclei, these ceramics demonstrate excellent properties.^4^, ^9^ Clinicians in Austria and Saudi Arabia widely adopted chairside CAD‐CAM systems (e.g., PrimeScan, Dentsply Sirona),^12^, ^13^ and patients favor the convenience of digital impressions.^14^, ^15^ Chairside CAD‐CAM lithium disilicate, like e.max CAD, has consistently demonstrated its clinical effectiveness, with survival rates over 95%.^16^ Due to its desirable esthetic^17^ and mechanical properties,^8^, ^11^ in comparison to other crown materials such as composite resin, zirconia, and metal‐ceramic, it has become a popular choice among clinicians. Long‐term evaluations endorse CAD‐CAM lithium disilicate's durability,^18^, ^19^ affirming its choice for posterior crown restorations in the present in vitro study.

Recent developments have introduced novel lithium disilicate ceramics blended 0.5‐micron needle‐like and 0.2–0.3 micron platelet‐like virgilite crystals within a zirconia glass matrix, requiring a notably shorter partial crystallization process of only 4.5 to 12 min at 760°C, compared to the traditional 20–31 min process,^20^, ^21^, ^22^, ^23^ further expanding the range of CAD‐CAM materials available to practitioners. One such material, CEREC Tessera by Dentsply Sirona, offers a range of shades and translucency options.^20^, ^21^ This partially crystallized material requires a glazing firing process at 760°C.^22^, ^23^ The choice of cement for CEREC Tessera and IPS e.max CAD crowns might not be influenced by crown thickness^21^ as the company recommends any type of cement (adhesive, self‐adhesive, or conventional) for both anterior and posterior crowns. Although Dentsply Sirona claims that this novel ceramic offers a high flexural strength of over 700 MPa, limited independent data is available.^20^, ^24^, ^25^, ^26^, ^27^, ^28^

Therefore, this study aimed to compare the fracture resistance of a chairside CAD‐CAM lithium disilicate with virgilite crowns for mandibular first molars with varying occlusal thicknesses (i.e., 0.80, 1.00, 1.20, and 1.50 mm). Also, this study compared the resistance of this novel material for prosthetic restorations to the traditional lithium disilicate with identical occlusal thickness (i.e., only in the 1.0 mm scenario). The first null hypothesis was that there is a significant difference among fracture resistance of the virgilite lithium disilicate crowns based on different thicknesses. The second null hypothesis was that there is no difference in fracture resistance of virgilite‐containing and traditional lithium disilicate posterior crowns with the same occlusal thickness.

## MATERIALS AND METHODS

### Specimen preparation

The sample size was calculated using G* Power analysis with an effect size of 0.25 or 0.5, significance level (*α* = 0.05), and power (0.8). Further analysis suggested that each group required a sample size ranging from 9 to 35. Considering previous publications that used 10–15 samples per group,^21^, ^29^ a sample size of 15 crowns per group was deemed appropriate for this study.

Four mandibular right first molar typodont teeth (1560 Dentoform, Columbia Dentoform, Lancaster, PA, USA) were prepared with standardized chamfer margins (1.00 mm) and varying occlusal reductions (0.8, 1.00, 1.20, and 1.50 mm). Digital impressions of the prepared teeth were obtained using a chairside CAD‐CAM system (CEREC Primescan, Dentsply Sirona, Charlotte, NC, USA). A total of 75 restorations were manufactured, categorized by thickness and material into five groups (*n* = 15 per group): CEREC Tessera crowns with 0.80 mm occlusal thickness, CEREC Tessera crowns with 1.00 mm occlusal thickness, CEREC Tessera crowns with 1.20 mm occlusal thickness, CEREC Tessera crowns with 1.50 mm occlusal thickness, and e.max CAD crowns with 1.00 mm occlusal thickness. Detailed information regarding the chairside CAD‐CAM dental ceramics used can be found in Table 1.

**TABLE 1:** Description of milled ceramic materials included in this study.

Ceramic brand	Manufacturer	Chairside crystallization requirements	Composition
CEREC Tessera^20^, ^21^, ^22^, ^23^	Dentsply Sirona (Charlotte, NC, USA)	Stand‐by temperature: 400°C; closing time: 3:30; heating rate: 60°C/min; firing temperature: 760°C; and holding time: 1:30 min.	57%−80% SiO2; 11%−19% Li2O; 0%−13% K2O; 0%−11% P2O5; 0%−8% ZrO2, 0%−8% ZnO; 0%−12% other oxides.
e.max CAD^6^, ^7^, ^8^, ^9^	Ivoclar Vivadent (Schaan, Liechtenstein)	Stand‐by temperature: 403°C; closing time: 6 min; heating rate: 90°C/min; firing temperature: 820°C; and holding time of 0:10 min.	SiO2: <78%, Li2O: <12% and coloring oxides: <12%.

The mandibular right first molar teeth were scanned using a laboratory scanner (D100, 3Shape, Copenhagen, Denmark), resulting in the production of 75 resin dies (Model Resin, Formlabs, Somerville, MA, USA) through a three‐dimensional (3D) printer (FormLab 3, Formlabs, Somerville, MA, USA). The CEREC Tessera crowns were subjected to glazing and sintering processes as per the guidelines set by the manufacturer. The firing protocols for both CEREC Tessera and e.max CAD crowns strictly followed the manufacturer's instructions. The Programat C2S furnace (Ivoclar Vivadent) was used consistently for both materials, allowing uniformity in terms of heating rate, holding time, cooling rate, peak temperature, vacuum settings, firing cycles, and atmospheric control throughout the processes. Thus, there were no variations in the firing protocols. Homogeneity testing, which evaluates a sample's compositional and characteristic uniformity, was not conducted in this study as it fell outside the research scope.

All restorations received appropriate intaglio surface treatment with 5% hydrofluoric acid application for 30 s (for CEREC Tessera) and 20 s (for e.max CAD), cleaning with Ivoclean (Ivoclar Vivadent) paste, and silanization with Monobond Plus (Ivoclar Vivadent). Printed resin dies underwent 38% etching with Total Etch (Ivoclar Vivadent) for 165 s, followed by rinsing and air‐drying. Cementation of restorations was accomplished using Multilink Automix resin cement (Ivoclar Vivadent) under a load of 200 g. It was light‐cured from occlusal, buccal, lingual, mesial, and distal for 20 s in each area and allowed to self‐cure for 6 min.

### Fracture assessment

In this study, the measured outcome was fracture resistance, quantified as the maximum stress a material can withstand without failure,^30^ usually expressed in megapascals (MPa) or Newtons per square millimeter (N/mm^2^). This critical property assesses the durability of dental restorations, which, if below the necessary threshold, are prone to fracture under typical chewing forces, necessitating additional clinical intervention.^31^, ^32^

**FIGURE 1:**
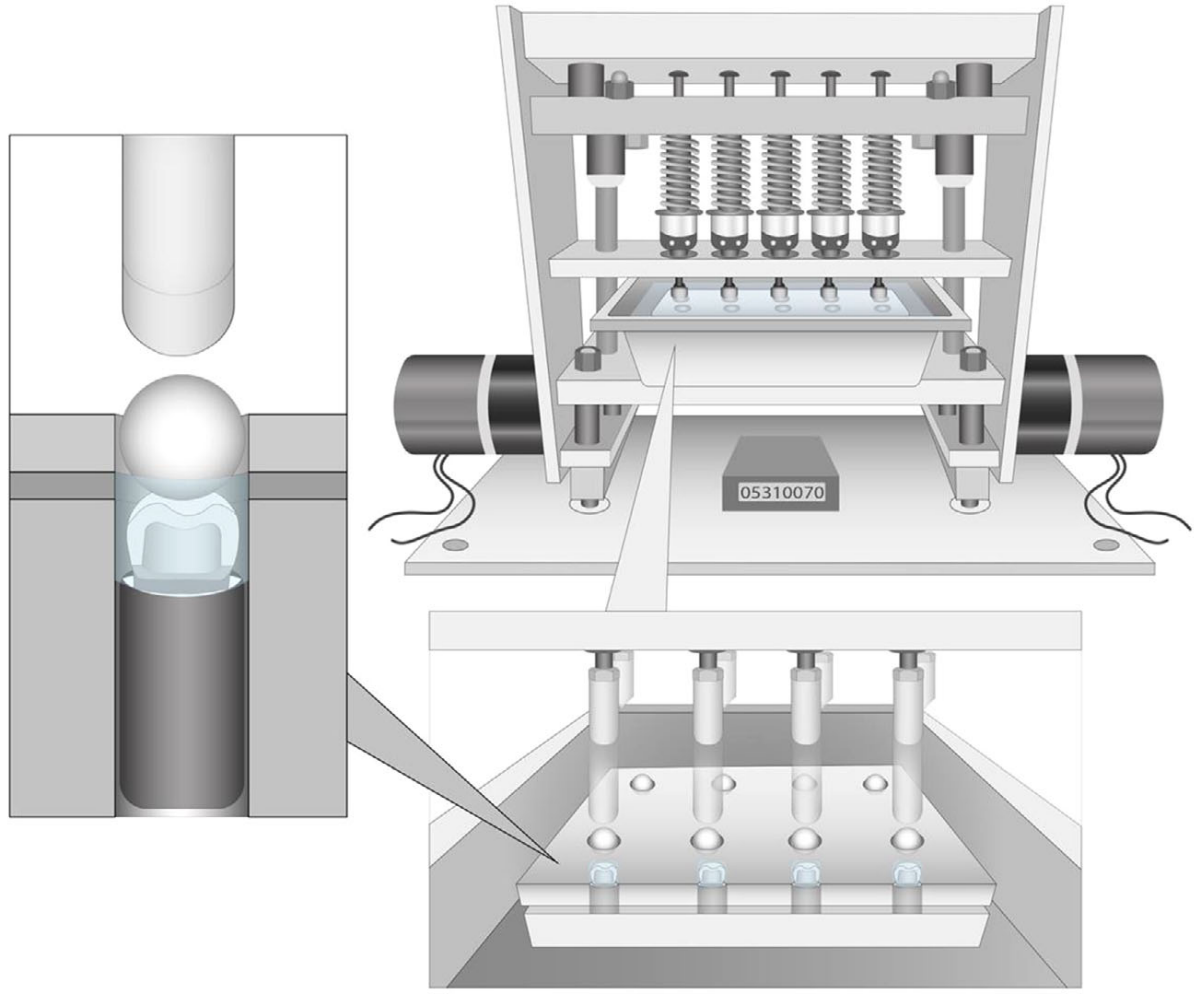
Schematic representation of the fatigue loading machine used for subjecting the specimens to cyclic loading before fracture testing.

The cemented restorations were securely positioned within a steel jig and subjected to cyclic loading using polyethylene balls (Delrin Dupont, Wilmington, DE, USA) for 5 million cycles at 1 Hz, applying a load of 275 N, all conducted in a controlled environment (distilled water) at 23°C (Figure 1). Subsequently, restorations were loaded until fracture, and load values were recorded in Newtons using an Instron 4204 testing machine (Norwood, MA, USA).

### SEM imaging

All specimens were visually assessed, and one specimen per group was chosen for scanning electron microscopy (SEM) images. The selected specimen exhibited patterns similar to all other specimens within the same group. Field emission SEM images were obtained by coating the fractured specimens with gold using a sputter coater (Quick Coater Type SC‐701, Sanyu Electric, Singapore) to ensure electrical conductivity. The images were captured at an accelerating voltage of 15 kV.

### Data analysis

The fracture resistance data were statistically analyzed using the independent‐sample Kruskal‐Wallis test (SPSS Statistics 25, IBM, Armonk, NY, USA) to assess the influence of crown thickness at a significance level (*α* = 0.05). Pairwise comparisons with Bonferroni correction were conducted to evaluate differences between novel and traditional ceramics of the same and different thicknesses.

## RESULTS

### Fracture test for restorations

The fracture resistance of novel chairside CAD‐CAM lithium disilicate with virgilite, with varying thicknesses, and traditional lithium disilicate is summarized in Table 2. The fracture resistance of lithium disilicate with virgilite (CEREC Tessera) posterior crowns varied with occlusal thickness. For instance, crowns with 1.5 mm occlusal thickness showed the highest mean resistance at 2119 N/mm^2^, followed by 1.20 mm at 1982 N/mm^2^, 1.00 mm at 1763 N/mm^2^, and thinner 0.80 mm crowns at 1144 N/mm^2^. Traditional lithium disilicate (e.max CAD) crowns at 1.00 mm had the lowest mean resistance, at 814 N/mm^2^. The fracture resistance values across different thicknesses and between the two ceramic materials had significant differences (Table 3).

**TABLE 2:** Fracture resistance of milled lithium disilicate containing virgilite and traditional lithium disilicate molar crowns.

Group	Material	Thickness	Number of samples	Load for fracture mean in N/mm^2^ (SD)
1	CEREC Tessera (Dentsply Sirona)	0.80 mm	15	1144 (280)^A,B,C^
2	CEREC Tessera (Dentsply Sirona)	1.00 mm	15	1763 (561)^D^
3	CEREC Tessera (Dentsply Sirona)	1.20 mm	15	1982 (457)^A,E^
4	CEREC Tessera (Dentsply Sirona)	1.50 mm	15	2119 (508)^B,F^
5	e.max CAD (Ivoclar Vivadent)	1.00 mm	15	814 (146)^D,E,F^

*Note*: Significant differences are indicated by the presence of the same uppercase superscript letter within the load for fracture column.

See Table 3 for detailed information on Bonferroni's pairwise comparisons.

Abbreviation: SD, standard deviation.

**TABLE 3:** Pairwise comparisons of the fracture resistance of milled ceramic molar crowns.

	Group 1 (CT 0.80 mm)	Group 2 (CT 1.00 mm)	Group 3 (CT 1.20 mm)	Group 4 (CT 1.50 mm)	Group 5 (EC 1.00 mm)
Group 1 (CT 0.80 mm)					
Group 2 (CT 1.00 mm)	0.069				
Group 3 (CT 1.20 mm)	0.004	1.000			
Group 4 (CT 1.50 mm)	0.003	1.000	1.000		
Group 5 (EC 1.00 mm)	0.930	0.001	<0.001	<0.001	

*Note*: Sequential Bonferroni significance.

Abbreviations: CT, CEREC Tessera; EC, e.max CAD.

### SEM observations

SEM analysis of the fractured specimens utilized a fractographic approach (Supplementary Figures 1 and 2). Thinner lithium disilicate restorations with virgilite, especially at 0.80 and 1.00 mm thicknesses, showed fewer, more distinct fracture lines. In contrast, those at 1.20 and 1.50 mm displayed complex crack patterns with notable irregularities, with the 1.50 mm variant uniquely revealing fracture lines in the resin die.

## DISCUSSION

A primary aim of this comparative in vitro study was to assess the fracture resistance of chairside CAD‐CAM lithium disilicate with virgilite posterior crowns of varying occlusal thicknesses. The findings supported the first hypothesis, indicating significant differences in fracture resistance among virgilite‐containing crowns with different occlusal thicknesses (Tables 2 and 3). Considering that the novel ceramic with 1.00 mm thickness demonstrated a resistance twice that of traditional lithium disilicate, the second null hypothesis, which suggested no difference in fracture resistance between the novel lithium disilicate containing virgilite and traditional lithium disilicate with the same occlusal thickness, was rejected.

Recent studies have investigated the fracture resistance of chairside CAD‐CAM dental ceramics. Findings indicate significant differences in fracture resistance based on occlusal thickness. A study on traditional chairside CAD‐CAM lithium disilicate e.max CAD crowns showed that thickness impacts fracture resistance, with 1.50 mm crowns withstanding 1540 N, 1.00 mm crowns 1162 N, and 0.80 mm crowns 980 N.^33^ Similarly, zirconia crowns displayed varying resistances: 2.0 mm crowns at 5934 N, 1.5 mm at 5540 N, decreasing progressively to 0.5 mm crowns at 1454 N.^34^ The present study extends these findings to novel ceramic materials,^20^, ^24^, ^25^, ^26^, ^27^, ^28^ affirming the trend of thickness‐related fracture resistance. It also fills a research gap by comparing novel virgilite‐reinforced and traditional lithium disilicate mandibular molar crowns, both 1.0 mm thick. In another study comparing virgilate‐lithium disilicate and standard lithium disilicate disc‐shaped specimens, virgilate‐lithium disilicate showed lower fatigue strength, reliability, and surface roughness, while both had similar hardness values.^5^

Total human chewing forces typically range from 100 N during regular chewing to 320 N during habitual occlusion.^35^ This study's findings reveal that the evaluated dental crowns displayed fracture resistance values between 814 and 2119 N, exceeding the maximum natural biting force. Therefore, it can be inferred that all tested restorations possess fracture resistance within clinically acceptable limits. The SEM results from this study align with another study examining traditional lithium disilicate (IPS e.max CAD) at thicknesses of 0.80, 1.00, and 1.50 mm.^33^ The findings indicate that the crowns with 0.80 and 1.00 mm thicknesses exhibited fewer and less visible crack lines compared to the 1.50 mm crowns. The increase in fracture complexity observed in the thicker crowns may be due to the higher bending forces they are subjected to. In another in vitro study,^5^ evaluating both lithium disilicates, fractures initiated from surface imperfections within the area of tensile stress concentration.

Certain limitations of this study should be considered. First, it concentrated exclusively on mandibular molar crowns; thus, subsequent investigations are advised to incorporate other tooth locations. Another limitation arises from the use of printed dies. While natural dentition would introduce more complexities and variables, such as the need to source a significant number of caries‐free molars, manual tooth preparation, and careful handling to prevent desiccation, the use of printed dies is a common practice in in‐vitro studies.^21^, ^29^, ^36^, ^37^ The use of a single‐load fracture test may not fully replicate the varied mechanical stresses in the dynamic oral environment. Consequently, the failure modes observed in these tests may not fully represent in vivo crown performance. Also, more extensive crack studies are recommended to understand better the fracture patterns. As a result, these findings warrant careful interpretation and further clinical studies are necessary before providing clinical recommendations.

## CONCLUSIONS

The fracture resistance of the novel chairside CAD‐CAM lithium disilicate molar crowns with virgilite is not significantly influenced by variations in occlusal thicknesses between 1 and 1.5 mm. This suggests that within this range, this occlusal thickness may not be a critical factor for clinicians to consider under high‐load conditions. The new ceramic material with an occlusal thickness of 0.80 mm displayed higher fracture resistance than traditional lithium disilicate ceramics, even at a greater thickness of 1.00 mm. While providing valuable information for material selection in posterior restorations, clinical recommendations require further research and validation.

## Supporting information

Supplementary Figure 1

Supplementary Figure 2
