# Supplementary figures and images for: Influence of occlusal thickness on the fracture resistance of chairside milled lithium disilicate posterior full‐coverage single‐unit prostheses containing virgilite: A comparative in vitro study
Source: J Prosthodont. 2024 May 24;34(8):852–8. doi: 10.1111/jopr.13870 (PMC12541292; doi:10.1111/jopr.13870)

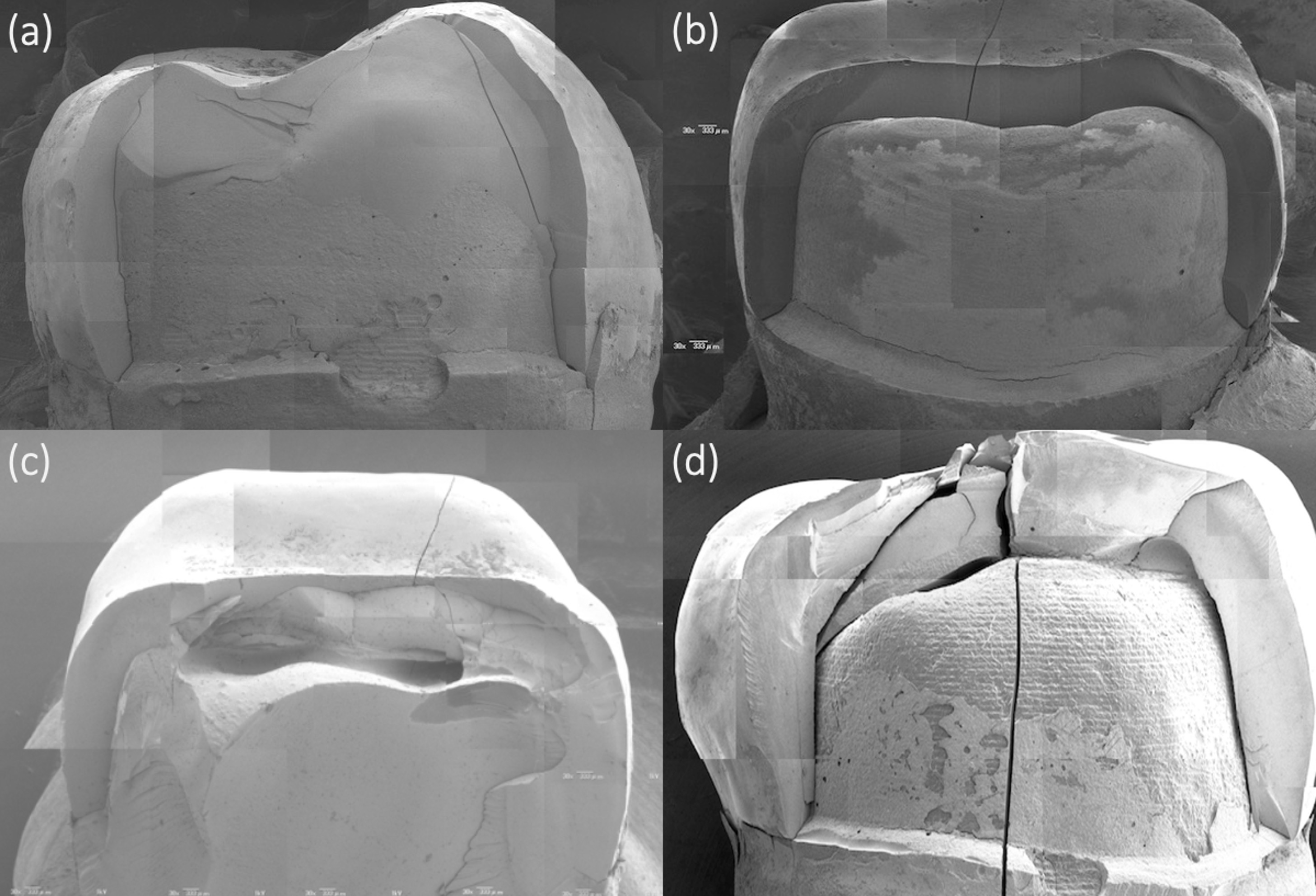

Supplement: Supplementary file 1 — Supplementary Figure 1 [file JOPR-34-852-s002.jpg]

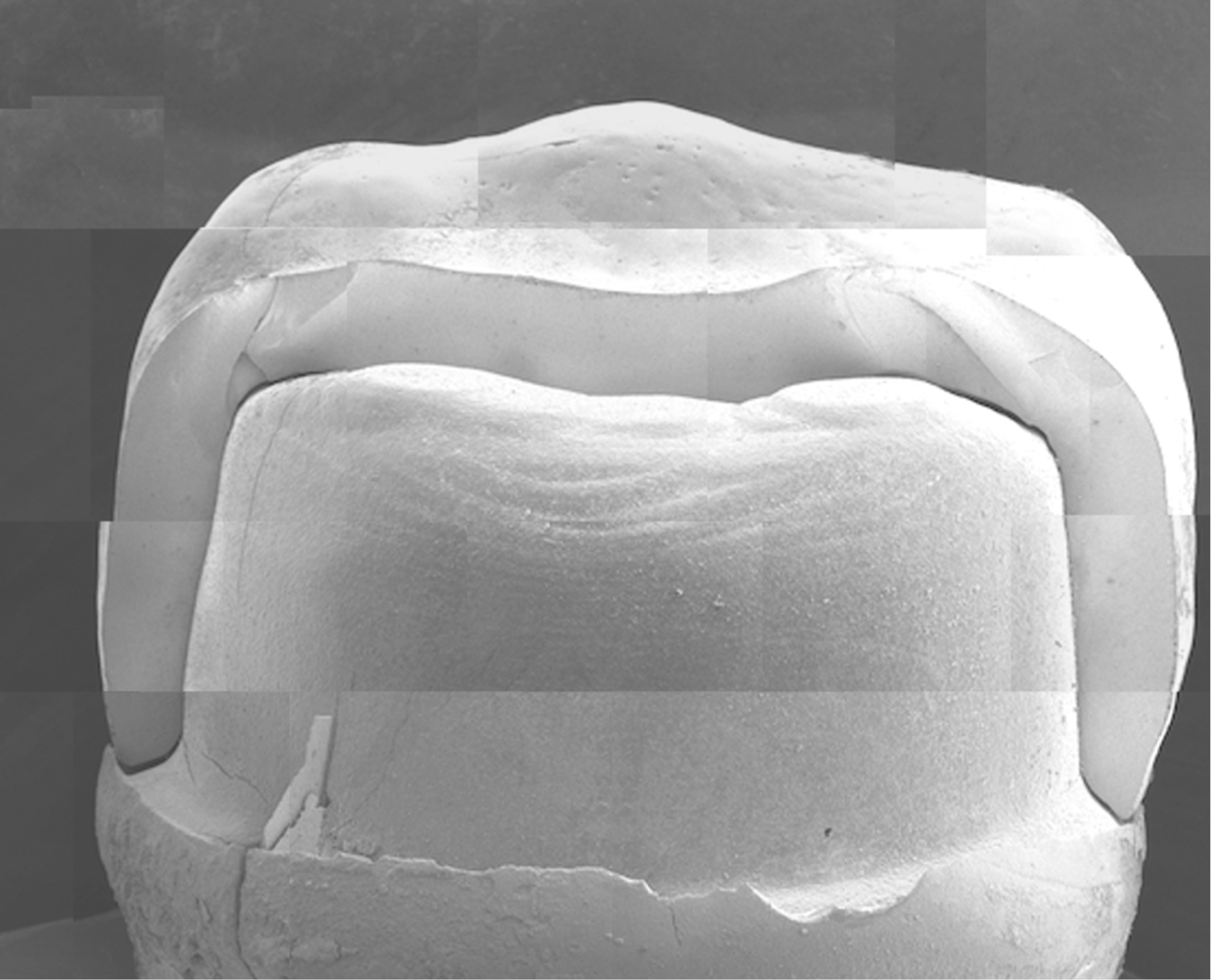

Supplement: Supplementary file 2 — Supplementary Figure 2 [file JOPR-34-852-s001.jpg]
